# Trust and Reciprocity: Are Effort and Money Equivalent?

**DOI:** 10.1371/journal.pone.0017113

**Published:** 2011-02-25

**Authors:** Iris Vilares, Gregory Dam, Konrad Kording

**Affiliations:** 1 Departments of Physical Medicine and Rehabilitation, Physiology, and Applied Mathematics, Rehabilitation Institute of Chicago, Northwestern University, Chicago, Illinois, United States of America; 2 International Neuroscience Doctoral Programme, Champalimaud Neuroscience Programme, Instituto Gulbenkian de Ciência, Oeiras, Portugal; French National Centre for Scientific Research, France

## Abstract

Trust and reciprocity facilitate cooperation and are relevant to virtually all human interactions. They are typically studied using trust games: one subject gives (entrusts) money to another subject, which may return some of the proceeds (reciprocate). Currently, however, it is unclear whether trust and reciprocity in monetary transactions are similar in other settings, such as physical effort. Trust and reciprocity of physical effort are important as many everyday decisions imply an exchange of physical effort, and such exchange is central to labor relations. Here we studied a trust game based on physical effort and compared the results with those of a computationally equivalent monetary trust game. We found no significant difference between effort and money conditions in both the amount trusted and the quantity reciprocated. Moreover, there is a high positive correlation in subjects' behavior across conditions. This suggests that trust and reciprocity may be character traits: subjects that are trustful/trustworthy in monetary settings behave similarly during exchanges of physical effort. Our results validate the use of trust games to study exchanges in physical effort and to characterize inter-subject differences in trust and reciprocity, and also suggest a new behavioral paradigm to study these differences.

## Introduction

Trust exists to some degree in all human interaction and is associated with a more healthy, egalitarian and productive society [1], [2], [3]. It can be defined as a positive expectation in the face of uncertainty emerging from social relations [4], [5]. Trust enables cooperative behavior, facilitates organization in both permanent and temporary work groups and is associated with higher job satisfaction, lower labor cost and larger profits [3], [5], [6], [7]. It can be seen in diverse types of interactions: people trust money to bankers, hoping that they won't run away with it; they trust their own home, letting complete strangers stay in their house and they also trust physical effort, for example by helping a friend move [4], [8]. Across such situations trust plays different roles and it seems important to understand commonalities and differences.

Trust is often justified as humans express reciprocity: they return helpful or harmful acts in kind, even though such behavior may come at a cost [9], [10]. As in trust, reciprocity is also expressed in different situations: in the examples above, the banker will work harder to maximize the earnings of the trustful investor, and a person that just stayed for free in someone else's house will more likely consider hosting as well [8]. If two people interact repeatedly, then not reciprocating but exploiting the partner has to be weighted against the cost of losing collaboration in the future [10], [11]. If, however, partners only interact once, then there is no direct risk of such retaliation. Nevertheless, even in one-shot interactions humans tend to reciprocate, while this behavior is much more difficult to find in other species [10]. Reciprocity in single encounters is of special interest for economists since in the current global market the traditional long-term repeated interactions between relatives or neighbors are being slowly replaced by one-time interactions between anonymous partners [10]. A better understanding of reciprocity in one-shot interactions in all its different contexts can then be of particular relevance to the current economy.

The importance of trust and reciprocity has been progressively recognized in the field of labor economics. Trust increases the ability of group members to work together [12] and promotes reciprocity [13]. It also seems to affect effort. For example, intensive control by a supervisor may lead to decreased work effort because it is sensed as an indication of distrust [14], [15]. Furthermore, trust within a group seems to affect their work effort, although the relationship between trust and effort is not very clear [16]. Some studies suggest that higher levels of trust can increase effort and efficiency towards the group task [12], [17]. Paradoxically, it has also been proposed that in some situations people with low trust will actually work harder when in a group, in order to compensate for the putative low performance of the co-workers [18]. Reciprocity, by its turn, not only reinforces trust but it can also increase employees working effort, functioning as an effective contract-enforcement device [19], [20]. These studies thus indicate that trust and reciprocity can affect effort, but how do they relate is still unclear.

In the context of behavioral economics, trust and reciprocity are often studied using trust games [2], [21], [22]. In such a game, one individual (the trustor) receives a given amount of money, and can choose how much of it to trust or invest. The trusted amount is then multiplied by some factor, for example three (symbolizing a return on social investment), and given to the other player (the *trustee*). The trustee can then decide how much of the proceeds to keep and how much to return to the trustor. The amount of money invested by the trustor is a measure of trust, and the amount repaid back by the trustee is a measure of reciprocity. In this way, trust games allow quantifying both the degree of trust as well as the degree of reciprocity.

Several studies have used trust games and the results have been contrary to what would be expected under the assumption of purely self-interested individuals, who act in order to maximize their own payoff [9]. In fact, if the trust game is played only once, then the optimal strategy of a purely self-interested trustee is to not reciprocate any money, and so the trustor, anticipating this, would invest nothing [2], [23]. Thus, for a one-shot trust game, the Nash equilibrium (the solution in which no player can increase their payoff unilaterally) is to neither trust nor reciprocate. Instead, it has been found that people do trust and reciprocate even at a cost to their gains [2], [21], [24]. However, these results were generally obtained using exchanges of money, and how this “monetary trust and reciprocity” can extrapolate to other contexts, such as effort, is still largely unknown [25].

Trust and reciprocity of effort may have different properties when compared to trust and reciprocity of money. Studies have suggested that trading effort instead of money can lead to different results, as it might increase the amount of cooperation [26] and can affect property rights [27]. Even in daily life this might be seen. For example, many people would easily give a day's worth of work to help a friend to move, but would not so easily offer them an equivalent amount of money [26]. There are thus indications that people are willing to entrust effort more than money. If trust and reciprocity differ between exchanges of money and effort, then caution is necessary when generalizing the results of the monetary trust games to the domain of effort.

In this study, we wanted to know how people trust and reciprocate effort in the context of a trust game. Specifically, we focused on physical effort, as it can be readily measured. Each of our 60 subjects participated in two computationally equivalent trust games, one involving physical effort and one involving money. We found that there were no significant differences in trust and reciprocity between effort and monetary conditions. These results hold even if we analyze only the first game of each subject. We also found that, across the two conditions, trust and reciprocity were strongly correlated. Finally, we observed that, although on average subjects reciprocated identically in both effort and monetary conditions, there was a much higher variability in the proportion reciprocated for the effort condition.

## Results

**Figure 1:**
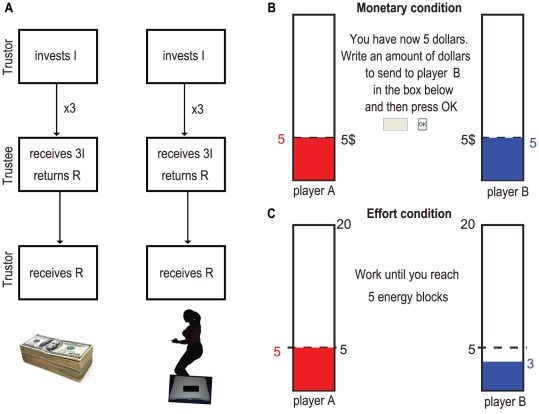
Experimental setup. **A**) Representation of the trust game used in this experiment. In each condition the trustor sends some amount I between 0 and 5 units, units which in the monetary condition (on the left) are US $ and in the effort condition (on the right) are energy blocks (EB). This amount is then tripled and sent to the trustee, who can then return some quantity R of this tripled amount (3I). In the effort condition both players have then to do the remaining squats in order to arrive to 20 EB. Each subject plays each condition only once. They keep the same role throughout the entire experiment but change partners between conditions. **B**) Example image presented on the computer screen when subjects were performing the monetary condition part of the experiment. In this phase, the trustor (“player A”) had just received the 5 $ show-up fee and had to decide how much to send to the trustee (“player B”). **C**) Example image presented on the computer screen when subjects were performing the effort condition part of the experiment. In this phase, the trustee (“player B”) was performing squats in order to arrive to 5 EB. While squatting, the correspondent bar (blue for the trustee, red for the trustor) was going up and the total number of EB possessed by the trustee was shown (as a blue number next to the trustee's rectangle). Once the trustee reached the 5 EB threshold the amount given by the trustor would be shown.

In this study we asked if trust and reciprocity differ between equivalent monetary and effort conditions of the trust game (see Fig. 1). In one condition subjects traded money (in units of US$) and in the other they traded effort, which was measured in energy blocks (*EB*). We considered the amount sent by the trustor as absolute trust and the amount sent back by the trustee as absolute reciprocity, or trustworthiness. We also considered relative trust, the amount sent by the trustor divided by the total amount available (in our case, the total amount the trustor had available was 5 $ or 5 *EB*) and relative reciprocity, the amount returned by the trustee divided by the total amount available (i.e. three times the amount sent by the trustor). Every subject participated in each condition once, changing partners between conditions. Subjects kept their role (trustor or trustee) throughout the experiment so that we could compare how they trusted or reciprocated across conditions.

**Figure 2:**
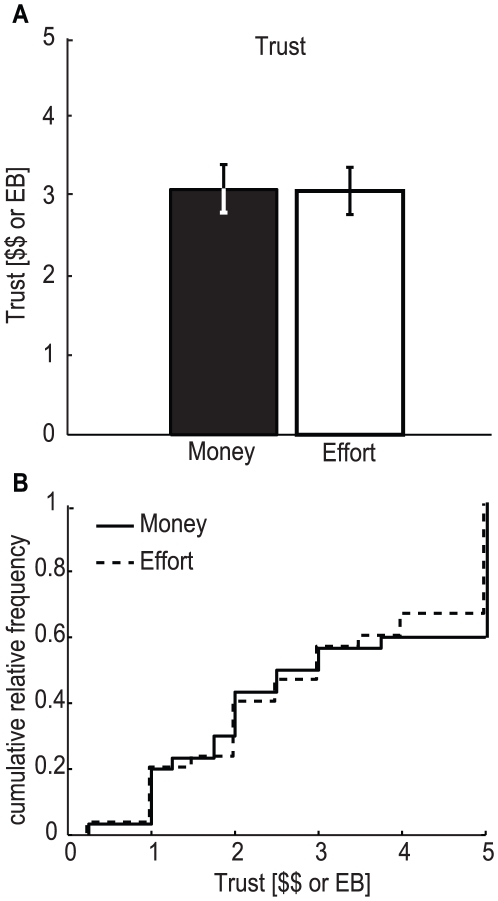
Quantity sent by the trustor, which is considered a measure of trust. **A**) Average trust in the monetary and the effort conditions (black and white, respectively). No significant difference in trust was found comparing the monetary and effort conditions (*p-val* = 0.9587; Wilcoxon signed-rank test; n = 30). Error bars represent standard error of the mean (s.e.m.). **B**) Cumulative distribution function for the amount sent by the trustor for the monetary (solid line) and the effort (dashed line) conditions. The distribution functions are not statistically different (*p = 1*, two-sample Kolmogorov-Smirnov test; n = 30).

We sought to test whether trust differs between monetary and effort conditions. We found that subjects displayed trusting behavior in both conditions (Fig. 2A), trusting μ_$_ = 3.1±0.3 $ in the monetary condition and similarly μ_w_ = 3.1±0.3 *EB* in the effort condition. Both averages were significantly different from zero, which is the Nash equilibrium for this game (*p-val*_$_ = 1.6×10^−6^ and *p-val*_w_ = 1.5×10^−6^, Wilcoxon signed-rank test). No significant difference in trust was found when comparing the monetary and effort conditions (*p-val* = 0.96; Wilcoxon signed-rank test; n = 30). Comparing the distribution functions (see Fig. 2B) also no difference can be found (*p-val = 1*, two-sample Kolmogorov-Smirnov test). This lack of difference between trust in the monetary and effort conditions surprised us, as we had expected subjects to trust more in the effort condition of the task.

We wondered if the lack of a statistical difference was due to an insufficient sample size. We therefore performed a power analysis asking which effect size we should have detected 90% of the time, at a α = .05 level of significance. We found that with the variance present in the data and the number of subjects used we should have been able to observe a difference if it had exceeded 15%. Therefore, a difference between both conditions, if existent, should be smaller than this value. Trusting behavior in our experiment, thus, seems very similar between the monetary and the effort conditions.

**Figure 3:**
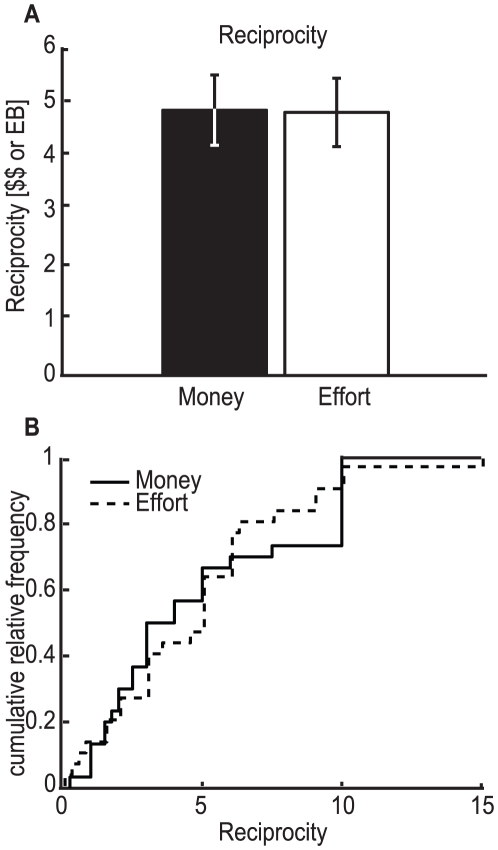
Quantity sent back by the trustee, which is considered a measure of (absolute) reciprocity. **A**) Average reciprocity in the monetary and the effort conditions (black and white, respectively). No significant difference was found comparing the monetary and effort conditions (p = 0.9519; paired t-test; n = 30). Error bars represent s.e.m. **B**) Cumulative distribution function for the amount returned by the trustee for the monetary (solid line) and the effort (dashed line) conditions. The distribution functions are not statistically different (*p* = 0.76, two-sample Kolmogorov-Smirnov test; n = 30).

We also wanted to know if reciprocating behavior differs between a monetary and an effort condition. We found that subjects reciprocated in both conditions (Fig. 3a), returning μ_$_ = 4.9±0.6 $ in the monetary condition and μ_w_ = 4.8±0.6 *EB* in the effort condition. The averages were again significantly different from zero, the Nash equilibrium (p-*val*_$_ = 1.6×10^−6^ and p-*val*_w_ = 2.5×10^−6^, Wilcoxon signed-rank test). The same is observed if we look at the relative reciprocity (μ_$_ = 0.49±0.03$ and μ_w_ = 0. 54±0.05 EB, *p-val*_$_ = 1.5×10^−6^ and *p-val*_w_ = 2.4×10^−6^, Wilcoxon signed-rank test). No significant difference in absolute reciprocity was found comparing the monetary and effort conditions (*p-val* = 0.95; paired t-test). Comparing relative reciprocity, there might be a trend for reciprocating more in the effort condition, but the difference is not significant (*p-val* = 0.27; paired t-test). Looking at the cumulative distribution functions of monetary versus effort reciprocity (see Fig. 3B) no significant difference can be found (*p-val* = 0.76, two-sample Kolmogorov-Smirnov test). A power analysis (as above) gives an upper limit of 14%, so any real difference, if it exists, should be smaller than that value. These results indicate that reciprocity does not differ significantly between the monetary and effort conditions, and in both cases subjects reciprocated more than predicted by Nash equilibrium.

**Figure 4:**
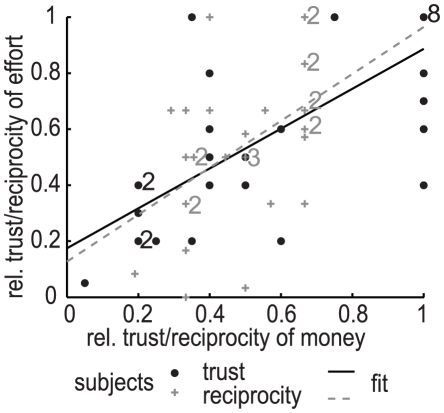
Relationship between a subject's relative trust or relative reciprocity in the monetary and effort conditions. Each trustor is represented by a black dot (n = 30) and each trustee is represented by a grey cross (n = 30). When dots or crosses overlap a small number is shown nearby, representing the amount of overlapping dots (in black) or crosses (in grey). The black solid line represents a linear regression of the relative amount trusted in the effort condition as a function of the relative trust in the monetary condition (β = 0.71, *p-val* = 1.6×10^−6^, r^2^ = 0.57). The grey dashed line represents a linear regression of the relative amount reciprocated in the effort condition as a function of the relative amount reciprocated in the monetary condition (β = 0.84, *p-val* = 6.9×10^−3^, r^2^ = 0.23).

We wanted to test if, at an individual level, a subject's behavior in the monetary condition was correlated to behavior in the physical effort condition. We designed the experiment so that each subject participated in one monetary and one effort condition in random order and thus we can analyze correlations across the conditions. We found a high positive correlation in subjects' trusting behavior between the monetary and effort conditions (r = 0.74, *p-val*<10^−5^, spearman correlation; see Fig. 4). We also found a significant but weaker correlation in reciprocating behavior (r = 0.39, *p-val* = 0.032 for absolute reciprocity; r = 0.48, *p-val* = 0.008 for relative reciprocity; spearman correlations, n = 30; see Fig. 4). Thus, subjects' behavior was positively correlated between conditions, with subjects that trusted or reciprocated more in a monetary condition tending to be more trusting/trustworthy in a physical effort condition.

To better understand how people reciprocate trust, we analyzed the correlations between subjects' investment and what they received back from the trustee. We found significant positive correlations between the amount subjects trust and what they receive back from the trustee, for both the monetary (r_$_ = 0.93, *p-val*_$_<10^−8^) and the effort (r_w_ = 0.73, *p-val*_w_<10^−5^) conditions. The high correlation between trusted and returned amounts can be expected, since more trust provides more money or energy blocks that can be returned. However, looking at the correlations between the relative values (relative trust with relative reciprocity), they are much weaker in the monetary condition (r_$_ = 0.38, *p-val*_$_ = 0.04), and disappear in the effort condition (r_w_ = −0.14, *p-val*_w_ = 0.45). Thus, subjects give back more if they receive more, although the relative reciprocity does not appear to depend strongly on the relative trust.

We also wanted to know how trustor's behavior in the second round was correlated to behavior of the trustee in the first round. We found a strong positive correlation (r = 0.73, *p-val*<10^−5^, n = 30, spearman correlation) between the absolute amount received in the first round and what is trusted in the second round. Thus, as it has been observed before [23], positive interactions in one round are correlated with cooperative behavior in the next.

To understand if this correlation is due to a causal influence of trustee's behavior in round one on trustor's behavior in round two, we constructed a multiple linear regression model in which both trust and reciprocity in round one are used as predictors of trust in round two. According to the model's fit, trust in the first round influences trust in the second (b = 0.58, *p-val* = 0.01), which would be expected if trusting is a character trait. We found that the behavior of the trustee in round one, on the other hand, had no significant influence (b = 0.14, *p-val* = 0.22), which would be expected if trustors take into consideration that they are playing with two distinct individuals. It appears that subjects begin the study with a certain level of trust, which is shared between the monetary and the effort conditions, and that they do not significantly update that level based on experience during the first round.

**Figure 5:**
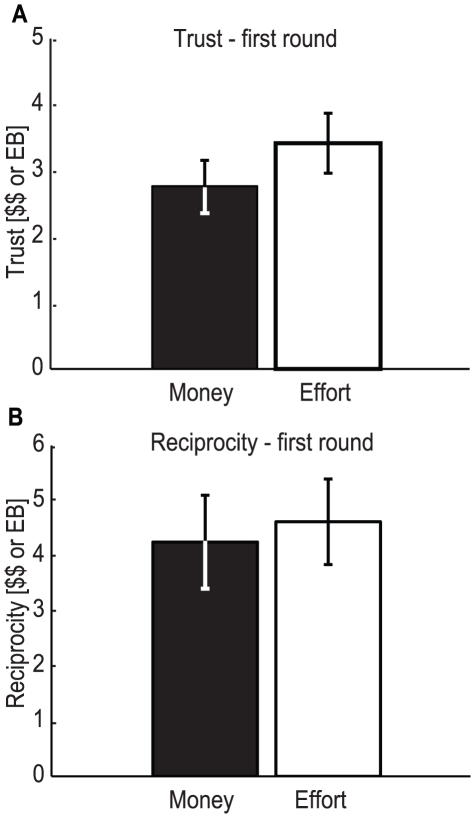
Trust and reciprocity values using only data from the first round. **A**) Average amount sent by the trustor (trust) in the monetary and the effort conditions (black and white, respectively), using only data from the first round. No significant difference in trust was found comparing the monetary and effort conditions (*p-val* = 0. 346; Wilcoxon rank-sum test, n = 15). **B**) Average amount reciprocated by the trustee in the monetary and the effort conditions (black and white, respectively), using only data from the first round. No significant difference was found comparing the monetary and effort conditions (p = 0.68; Wilcoxon rank-sum test, n = 15). Error bars represent s.e.m.

It is possible that the similarity across rounds occurred because subjects simply decided to behave in the second round in the same way they did during the first. To rule out this hypothesis we compared behavior using only the first round of the game. In this way, every subject only contributes one independent data point (see Fig. 5). Subjects trusted μ_$_ = 2.8±0.4 $ and μ_w_ = 3.4±0.5 *EB* and reciprocated a total of μ_$_ = 4.3±0.8 $ and μ_w_ = 4.6±0.8 *EB* (values significantly different from zero, *p-val* = 6.1×10^−5^ for all cases, Wilcoxon signed-rank test, n = 15). The relative returns follow the same tendency (μ_$_ = 0.47±0.04 $ and μ_w_ = 0.50±0.06 *EB*; *p-val* = 6.1×10^−5^). Comparing the monetary and effort conditions, there was a tendency for higher trust and reciprocity in the effort condition, but this difference is also non-significant (p = 0.35 for trust and p = 0.68 or p = 0.69 for absolute and relative reciprocity; Wilcoxon rank-sum test, n = 15). Thus, the observed similarity across conditions does not seem to be a result of the repeated nature of the experiment.

To test if there were any gender differences in our results, we compared male and female behavior in the different conditions. For both the monetary and the effort conditions, we found no significant difference between males and females in both the amount trusted (*p-val*_$_ = 0.45 and *p-val*_w_ = 1; Wilcoxon rank-sum test; n_♀_ = 17 and n*_♂_* = 13) and the amount reciprocated (*p-val*_$_ = 1 and *p-val*_w_ = 0.54 for absolute reciprocity; *p-val*_$_ = 0.37 and *p-val*_w_ = 0.58 for relative reciprocity ; Wilcoxon rank-sum test; n_♀_ = 18 and n*_♂_* = 12). Also, analyzing separately males and females, we found no differences between behavior in the monetary and the effort condition, in both the amount trusted (*p-val*_♀_ = 0.17 and *p-val_♂_* = 0.45; Wilcoxon signed-rank test) and the amount reciprocated (*p-val*_♀_ = 0.59 and *p-val_♂_* = 0.46 for absolute reciprocity; *p-val*_♀_ = 0.41 and *p-val_♂_* = 0.38 for relative reciprocity; Wilcoxon signed-rank test). This lack of gender differences suggests that the similarity between behavior in the monetary and the effort conditions does not seem to depend on the person's gender.

**Figure 6:**
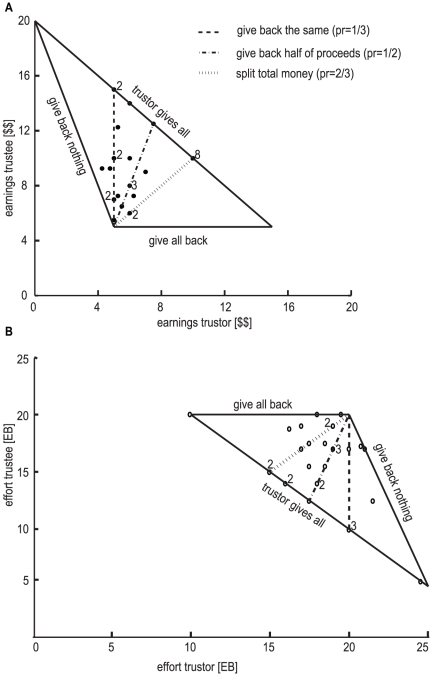
Distribution of joint earnings or effort. **A**) Representation of trustor's and trustee's earnings in the monetary condition(figure similar to the one presented in [21]). Each subject pair/output of a round is represented by a filled dot. When dots overlap a small number is shown near the dot, representing the amount of overlapping dots. The outer triangle shows the set of possible earning pairs. The lines represent different possible relative reciprocity values (how much of the tripled amount sent by the trustor the trustee sent back): (1) if the relative return (*p_r_*) is equal to zero (“give back nothing” line), it means that the trustee keeps all the money; (2) when *p_r_* = 1/3, the trustee sends back the exact amount trusted(dashed line), so the trustor neither wins nor loses. Points that fall to the left of this line indicate that the trustor lost something by trusting, while points at the right of the line indicate the opposite; (3) if *p_r_* = 1/2, the trustee decides to split the tripled investment in half (dashdot line); (4) when *p_r_* = 2/3$, the trustee splits in half the total earnings, inclusive of show-up fees (dotted line); (5) finally , when *p_r_* = 1$ the trustee returns the total of the tripled investment, which is the maximum he can return(“give all back” line). Dots more near the line confluence vertex of the triangle indicate that the trustor showed lower trust, while dots more near the “trustor gives all” line represent high trust by the trustor. A total number of n = 30 subject pairs is represented. **B**) Analogous figure to the one represented in *(A)*, but in which what is represented is the total effort (number of energy blocks that each player had to perform throughout the entire experiment). The triangle here is inverted since increasing trusts decreases the total effort necessary to finish the task (arriving at 20 EB). A total number of n = 30 subject pairs is represented.

To better understand the strategies used, we graphically analyzed subjects' decisions (Fig. 6). We observed more variability in the strategies employed in the effort condition. For example, in the effort condition subjects sometimes returned everything or nothing, neither of which happened in the monetary condition. In fact, in the monetary condition the vast majority of the trustees (93%) reciprocated between 1/3 (return exactly what was trusted) and 2/3 (split total earnings), while in the effort condition this percentage, although still high, decays to 70%. All trustors sent something to the trustees, almost all the trustees returned something, and the vast majority of the trustees (93% in the monetary, 87% in the effort condition) returned the same or more than the trustor sent. In several cases, (2 in the effort, 8 in the monetary condition) the trustor sent the entire show-up fee to the trustee, and the trustee returned 2/3 of it back – this point may be considered fair and efficient, since it maximizes the total money/*EB* to be shared and it divides it equitably. Most subjects appear to have followed simple decision-rules in both conditions, and the strategies employed in the effort condition seem more variable.

**Figure 7:**
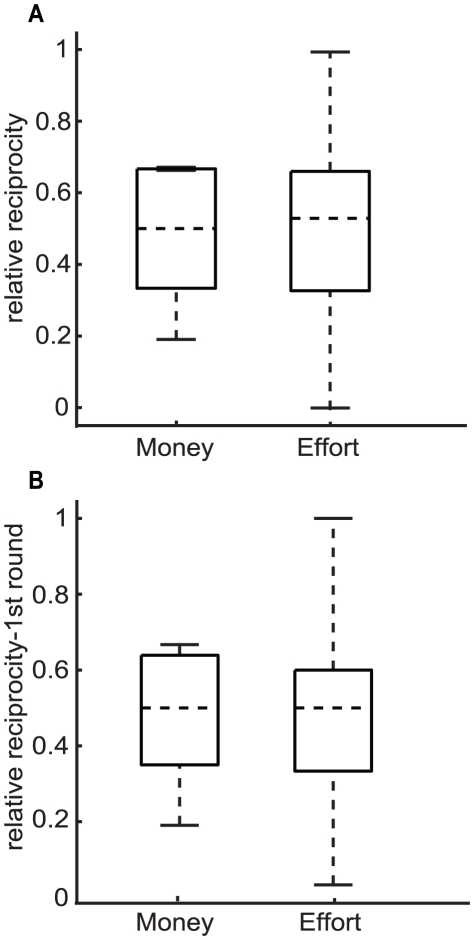
Box plots of the relative reciprocity obtained for the monetary and effort conditions. In **A**) all data was used (n = 30), while in **B**) just the data concerning the first experiment made by each subject was used (n = 15). The middle horizontal dashed line represents the median (50^th^ percentile), the lower horizontal solid line represents the minimum; the bottom and top of the box are the percentile 25^th^ and 75^th^, and the upper horizontal solid line represents the maximum of the data. Note that when no upper black line can be seen it is because the 75^th^ percentile and the maximum are the same.

To further analyze if the behavior is indeed more variable in the effort condition, we computed the dispersion patterns in both conditions (Fig. 7). Relative reciprocity, although its average does not differ across conditions (recall Fig. 3), has more variance in the effort condition. Testing for a difference in the variances gives a significant p-value (p = 0.002, paired-variance test, n = 30). A similar trend can be observed when looking only at the first round (Fig. 7 B), although the result is not significant (p = 0.19, Bartlett's variance test, n = 15). Thus, when subjects traded effort they showed a significantly higher variability of relative reciprocity values.

## Discussion

Here we analyzed if trust and reciprocity differ between monetary and effort conditions. Our original hypothesis, based on everyday observations, was that subjects in the effort condition would trust and reciprocate more. However, we found that across the monetary and the physical condition subjects showed the same level of trust and reciprocity. Moreover, subjects' behavior across conditions was highly correlated. The only significant difference was in the variability of behavior, with subjects showing a wider range of relative reciprocity values in the effort condition.

Subjects in both conditions trusted and were trustworthy, sending on average more than half of their monetary earnings or accumulated effort to the other player. The levels of trust and reciprocity observed in the monetary condition are similar to those of analogous monetary experiments reported in the literature [21], [28]. Interestingly, these trusting and reciprocity values were obtained not only when subjects traded money but also when they traded physical effort, suggesting that people's tendency to trust and reciprocate also applies to physical effort decisions.

Previous studies in movement effort decision-making have found some similarities between the decisions made in effort contexts and the ones generally made in monetary settings. For example, in a study in which subjects had to move in order to receive rewards or avoid punishment [29], subjects displayed behaviors such as loss aversion and diminishing returns, phenomena typically described in monetary settings [30]. Here, we show that this similarity between movement and economic decisions also extends to social decisions.

Recent research has examined trust and reciprocity in an experiment in which physical effort was involved [27]. In a one-shot trust game, subjects had to expend effort, specifically crack walnuts, in order to receive money, which they could then use in a trust game. Both trustors and trustees tended to give more money to subjects that had worked – it is as if the work had resulted in “property rights”. In this experiment only money was traded and the authors focused on how effort affected the interactions in a trust game, while in our study we asked how the nature of traded units affects behavior and therefore we could ask if trust and reciprocity are shared across these domains.

Our results show, even on the individual level, that trust and reciprocity are very similar between the monetary and the effort conditions. This can indicate that trust and reciprocity may be character traits. This idea is supported by previous studies, which also reported high correlations between cooperative behaviors in a trust game paradigm (meaning trust and trustworthiness) and specific personality traits [28], [31], [32], [33]. There has been also some indication that part of this cooperative behavior in trust games can be heritable, with monozygotic twins behaving in a more similar way when compared to dizygotic twins [22]. Our results contribute to the view that trust and reciprocity are true character traits.

Why would trust and reciprocity be shared across monetary and effort conditions? Trust and reciprocity have governed social interactions over evolutionary timescales, and it was thus suggested that they could tap into ancient neural systems involved in social cooperation or even directly into reward pathways [11], [23], [34], [35], [36]. Since cooperation for joint effort is older than monetary cooperation, we should expect an increased reliance on these primitive pathways. We thus suggest that the behavioral correlation observed between monetary and physical effort conditions may be a result of shared neural substrates. In future studies, this hypothesis could be tested experimentally using neuroimaging approaches.

Another interesting future study to perform would be to check the specific effects of property rights on behavior in the money and effort conditions. In our experiment, while in the effort condition people worked to arrive at 5 energy blocks that they then traded, in the monetary condition people received the $5 as a show up fee without having to work for it. This created a potential difference in the property rights people could feel about the trading units. Surprisingly, this potential difference between conditions did not result in a measurable difference in behavior. It would be interesting to test the effects on behavior when property rights are elicited for the monetary version, for example by having subjects solve a given number of puzzles in order to get the initial $5. We hypothesize that this could create an even higher similarity between behavior in the monetary and the effort conditions.

The only difference we found across the conditions was that relative reciprocity is more variable in the effort condition. This difference can be due to a higher variance of fitness levels across the subject pool compared to variance of average earnings, although this is unlikely the only cause as this higher variability is not exhibited in trusting behavior. It is also possible that reciprocity of effort has less stringent social norms. Given that money is typically easily quantified and carries very strong emotional values [37], there can be specific social norms on what someone should reciprocate monetarily, but these social norms may be less stringent when it comes to effort retribution. These hypotheses could be tested in future experiments by changing the pool of participants as well as the social framing of the experiment.

The fact that the behavior in both effort and monetary conditions was similar and correlated has two major methodological implications. First, it validates the use of the typical monetary trust game as an effective tool to study trust and reciprocity. Second, it opens the possibility of studying trust and reciprocity using physical effort tasks. Effort based tasks may have a number of advantages. One advantage is that such experiments may be done cheaply over a wide range of investments (from a single squat to hours of hard workout). Furthermore, it can be a good alternative when comparing trust games across different countries, with different monetary units and/or different purchasing power, as the value of each energy block is less likely to be influenced by the country from which a person is from. Finally, it can allow for a better sampling of the population, as it is easier (and probably cheaper) to get a wider coverage of the population's physical effort cost functions than of the monetary cost functions, and can thus offer us a bigger and more representative set of behaviors. The physical effort condition that we introduced here may be seen as a new tool to study trust and reciprocity, complementing the use of monetary trust games.

## Materials and Methods

### Participants

A total of 60 healthy volunteers (35 females, 25 males; age 30±9 years) participated in the experiment. All experimental protocols were performed in accordance with federal guidelines and the Northwestern University's policy statement on the use of humans in experiments. Informed consent was obtained from all participants.

### Experimental procedure

Each experimental session consisted of a trust game presented in 2 conditions, monetary and physical effort. All subjects in the same session experienced the same sequence of events. The choice to start with the effort or the monetary condition was pseudorandom, so that half of the volunteers started with one condition, and the other half started with the other, to avoid any potential priming effects. Each subject played both conditions of the trust game once. Subjects were randomly assigned to be either player A (trustor) or player B (trustee) and kept that role throughout the experimental session. In order to minimize potential multi-round effects, subjects were randomly matched for the first condition of the experiment and changed partners between conditions. Players with the same role were placed in the same room, and were informed that they would only be playing with subjects that were in another room. They were instructed to not discuss strategies with one another. Furthermore, they were also informed that the person with whom they were playing would change between experiments. Subjects were given no information that enabled them to identify their partner and were also asked to keep their decisions private. They received written instructions (see Appendix S1). The instructions and the computer screen were phrased as neutral as possible; words like “trust”, “cooperation”, “competition” and “opponent” were avoided. An experimental session (including instructions, both experiments and waiting time) averaged 60 minutes. Earnings averaged $18 and ranged between $14 and $25.

### The Game

In the *monetary condition*, both player A (trustor) and player B (trustee) received 5 US dollars ($5) as a show-up fee. Player A then decided to send all, none or some (in multiples of $0.25) of the show-up fee to player B. The amount sent to player B was tripled. Player B then decided how much of that money to send back to player A and how much to keep (see Fig. 1 A). In the *effort condition*, both player A and player B had to perform squats while standing on a 4-sensor force-plate until each of them reached 20 “energy blocks” (EB). Initially both player A and player B had to perform squats until a total of 5 energy blocks was reached. Player A then had the opportunity of sending a portion of the 5 energy blocks (in multiples of 0.25) to player B. The amount of energy blocks sent to player B was tripled. Player B then decided how many energy blocks to return to player A (see Fig. 1 A). Each player had then to perform the remaining squats in order to reach the 20 energy blocks required for the task. Each player received $10 for completing this part of the experiment.

### Data acquisition

Participants wrote their decisions in a box on a computer screen. At each point of time, the computer screen showed two rectangles, one at the left side of the screen with the amount of dollars/energy blocks possessed by player A (trustor) and one at the right side with the amount of dollars/energy blocks possessed by player B (trustee). Furthermore, a color-code was given: red for the dollars/energy blocks earned by the trustor and blue for the ones earned by the trustee (see Fig. 1 B and C). Subjects' responses were recorded using Matlab. For the effort experiment, data was collected using a 4-sensor force-plate (Nintendo Wii™ Balance Board, recorded at 500 Hz). By performing squatting movements with their body, subjects produced forces, which were then translated to Energy Blocks.

An energy block (EB) is a multiple of the work produced by that subject (W) per unit of mass (m):
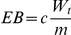
Where *c* is a constant, in this case *c = 1/4*. Work was defined at each point of time *t* based on the forces recorded via:
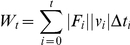
Where *F_i_* is the average force obtained by the 4-sensor force plate (in *Newtons*) at each iteration *i*, *v_i_* is the velocity and *Δt_i_* is the amount of time that passed between *i-1* and *i* (which was, on average, about 0.035 s).

## Supporting Information

Appendix S1 **Instructions for participants.**
(DOC)
