# Supplementary material for: Trust and Reciprocity: Are Effort and Money Equivalent?
Source: PLoS One. 2011 Feb 25;6(2):e17113. doi: 10.1371/journal.pone.0017113 (PMC3045406; doi:10.1371/journal.pone.0017113)
Supplement: Appendix S1 — Instructions for participants. (DOC) [file pone.0017113.s001.doc]

SUPPORTING INFORMATION

INSTRUCTIONS FOR PARTICIPANTS

Thank you for participating in our economics of movement experiment. Please read the following instructions carefully.

You will perform two different experiments: a work experiment and a monetary experiment. The order in which you will perform them will be decided at random.

In each experiment you will be paired with a **different person, whom you will never meet and will remain in a separate room.**

In both experiments, you will be performing the role of the: *Player A / Player B*

The monetary experiment proceeds as follows. Both *Player A* and *Player B* receive 5 US dollars ($5) as a show-up fee. The *Player A* will then decide to send all, none or some (in multiples of $0.25) of the show-up fee to the *Player B*. The amount sent to the *Player B* will be tripled. For example, if the *Player A* sends $1, the *Player B* will receive $3. The *Player B* will then decide how much of that money to send back to the *Player A* and how much to keep. Note that the amount *Player B* returns to *player A* is not tripled.

In the work experiment, both *Player A* and *Player B* have to work, *i.e.*, to perform squats while standing on a *wii fit board*, until they reach 20 “energy blocks”. The amount of energy used to squat will be converted into “energy blocks”. Note that larger squats will translate into more energy blocks. Initially both *Player A* and *Player B* will have to perform squats until a total 5 energy blocks are reached. *Player A* will then have the opportunity of sending all, none or some (in multiples of 0.25) of the 5 energy blocks to the *Player B*. The amount of energy blocks sent to the *Player B* will be tripled. For example, if the *Player A* sends 1 energy block, the *Player B* will receive 3 energy blocks. *Player B* will then decide how many energy blocks to send back to *Player A* and how many to keep. Note that the amount *Player B* returns to *player A* is not tripled.

After all energy blocks have been sent between players, starting with the number they have at that time, each player will have to reach a total of 20 “energy blocks” to receive $10.

These experiments are designed so that no participant will know with whom they have been paired with, nor will they know the personal decision of the other participants. Since your decision is private, we ask you not to tell anyone else about your decision either during, or after the experiment.

You will participate in both the monetary and the work experiment. When the experiments are switched, **the person you are paired with also changes**. Your role however (*Player A* or *Player B*) will remain the same for both experiments.

You will be asked to perform the experiment in front of a computer screen. On the screen you will see two rectangles: one labeled “*Player A*” (on the left part of the screen) and one labeled “*Player B*” (on the right part of the screen). The number of dollars/energy blocks is represented by the height of the colored filling of the rectangle, and indicated by the number next to the rectangle.

We will ask you to switch rooms in between experiments or turns so that you will not meet the players you are paired with.

Monetary Experiment

At the beginning of the experiment a $5 show-up fee is given to each player. The experiment begins when *Player A* decides how much money to send to the *Player B*. *Player B* will receive this value tripled. *Player A* then writes the amount to be sent in a box on the screen, presses *“ok”*, puts that amount in an envelope and pockets the remaining money. Example: (1) Put $1 in the envelope, and pocket $4. (2) Put $4.5 in the envelope, and pocket $0.5. These are examples only. The actual decision is up to each person.

The envelope is given to the experimenter and *Player A* leaves the room. The experimenter will then triple the amount of money present in the envelope.

*Player B* then enters the room and receives the $5 show-up fee. This money is immediately pocketed. *Player B* then presses a keyboard key to see how much money *Player A* sent, and receives the envelope with that amount already tripled. *Player B* must then decide how much money to send back to *Player A*, writes that number in a box on the screen and presses *“ok”*. Amounts sent must be multiples of $0.25. *Player B* then leaves that amount in the envelope, pockets the remaining money and exits the room. For *Player B*, the monetary experiment is now over. We ask you to wait in a designated room if you still did not perform the work experiment, or to leave if you already did both experiments.

Finally, *Player A* enters the room again, reads on the screen how much *Player B* sent back, gets the envelope and pockets the money. The monetary experiment is now over, and *Player A* also leaves the room. We ask you to wait in a designated room if you have not performed the work experiment, or to leave if you already did both experiments.

Work Experiment

In the work experiment *Player A* is brought to the experimental room. *Player A* must perform up and down body movements (“squats”) while standing on a *wii fit board*, until reaching 5 “energy blocks”. Each squat will translate into a given number of “energy blocks”. Note that larger movements will translate into more work, thus, more energy blocks. **Jumping is not allowed.** When 5 energy blocks are reached, *Player A* then has to decide how many energy blocks to send to *Player B*. It has to be a number between 0 and 5, and multiple of 0.25. *Player A* will then write the value to be sent in a box on the screen and press “ok”. *Player B* will get this value tripled. This number is then taken away from the energy blocks *Player A* had. Example: (1) Send 1 energy block, and remain with 4 energy blocks. (2) Send 4.5 energy blocks, and remain with 0.5 energy blocks. These are only examples. The actual decision is up to each person. *Player A* leaves the room.

*Player B* then enters the room. First, *Player B* also must perform squats until 5 energy blocks are reached. After reaching the 5 energy blocks, *Player B* presses a keyboard key to see how many energy blocks *Player A* had sent, and receives them tripled (represented in red on the screen). *Player B* must then decide how many energy blocks to send back to *Player A*, writes that number in the box on the screen and press *“ok”*. Amounts sent must be multiples of 0.25. *Player B* then performs the remaining squats necessary to reach 20 energy blocks. After reaching the 20 energy blocks, *Player B* receives $10, pockets it, and leaves the room. For *Player B*, the work experiment is now over. We will ask you to wait in a designated room if haven’t already performed the monetary experiment, or to leave the building if you have completed both experiments.

Finally, *Player A* enters the room again, learns how many energy blocks *Player B* sent back (represented in blue in the screen) and performs the remaining squats necessary to reach 20 energy blocks. After reaching 20 energy blocks, *Player A* receives $10, pockets it, and leaves the room. We will ask you to wait in a designated room if you have not performed the monetary experiment, or to leave the building if you have completed both experiments.

Player A

Player A

sends I

receives 3I

returns R

x3

Player B

receives

R

Player A
